# Treatment of euvolemic hyponatremia in the intensive care unit by urea

**DOI:** 10.1186/cc9292

**Published:** 2010-10-14

**Authors:** Guy Decaux, Caroline Andres, Fabrice Gankam Kengne, Alain Soupart

**Affiliations:** 1 Research Unit on Hydromineral Metabolism, Department of General Internal Medicine, University Erasme Hospital, ULB, Route de Lennik 808, Brussels, B-1070, Belgium; 2 Department of Internal Medicine, Tubize-Jolimont Hospital, Avenue de Scandiano 8, Tubize, B-1480, Belgium

## Abstract

**Introduction:**

Hyponatremia in the intensive care unit (ICU) is most commonly related to inappropriate secretion of antidiuretic hormone (SIADH). Fluid restriction is difficult to apply in these patients. We wanted to report the treatment of hyponatremia with urea in these patients.

**Methods:**

Two groups of patients are reported. The first one is represented by a retrospective study of 50 consecutive patients with mild hyponatremia treated with urea. The second group is presented by a series of 35 consecutive patients with severe hyponatremia acquired outside the hospital (≤ 115 mEq/L) who where treated by isotonic saline and urea (0.5 to 1 g/kg/day), administered usually by gastric tube.

**Results:**

In the first group with mild hyponatremia (128 ± 4 mEq/L) the serum sodium (SNa) increased to a mean value of 135 ± 4 mEq/L (*P *< 0.001) after two days of urea therapy (46 ± 25 g/day), despite a large fluid intake (> 2 L/day). The mean duration of urea therapy was six days (from 2 to 42 days). Six patients developed hyponatremia again once the urea was stopped, which necessitated its reintroduction. Six patients developed hypernatremia (maximum value 155 mEq/L). In the second group, SNa increased from 111 ± 3 mEq/L to 122 ± 4 mEq/L in one day (*P *< 0.001). All the patients with neurological symptoms made a rapid recovery. No side effects were observed.

**Conclusions:**

These data show that urea is a simple and inexpensive therapy to treat euvolemic hyponatremia in the ICU.

## Introduction

In the intensive care unit, hyponatremia occurs frequently and is associated with an increased mortality [1-4]. It is mostly related to the presence of inappropriate antidiuresis due to an excess of ADH. Management of this condition usually implies water restriction. This is of poor applicability in patients requiring multiple intravenous medications and/or nutritional support. Recently a new class of drugs antagonizing the V2 receptor (V2RA) has been developed: the vaptans [5-9].

Two of them are already available on the market: Conivaptan for the intravenous route [10-16] and Tolvaptan for the oral route [17]. The present data recall that urea is an effective and easy therapeutic choice to correct hyponatremia related to SIADH [18-21] with special attention for patients in the intensive care unit. The main criticism to the use of urea orally is its taste; this is not a problem in the intensive care unit as it is usually administered by gastric tube or intravenously. No prospective data comparing V2 antagonists to urea are available. We present a large retrospective series of patients with moderate or severe hyponatremia treated with urea and shows that its use is a easy, save and inexpensive treatment.

## Materials and methods

### Study I - Moderate hyponatremia (120 to 134 mmol/L)

We analyzed the charts of 50 consecutive patients treated with urea in the intensive care unit.

**Figure 1:**
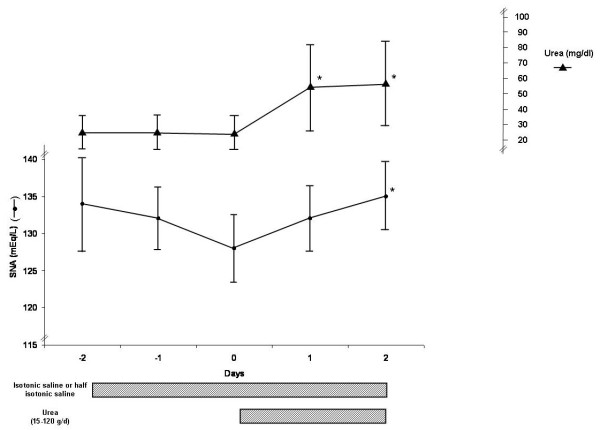
Evolution of SNa and blood urea in 50 patients before and after urea therapy.

Some serum parameters two days before and the first two days during urea therapy are presented (Figure 1). In 10 patients, urine parameters and balance data were also available (see Table 1). All the patients were receiving isotonic or half isotonic saline solutions before urea administration.

**Table 1:** Evolution of some blood and urine parameters in 10 patients with mild hyponatremia treated by 45 g urea/daily at least during three days

	***Day -1***	***Day 0***	***Day 1***	***Day 2***
S Urea (mg/dL) N < 40	25 (± 10.1)	25 (± 9.5)	60 (± 25.9)*	67 ± 29.7*
S Creat (mg/dL) N < 1.1	0.5 (± 0.1)	0.5 (± 0.1)	0.5 (± 0.1)	0.5 (± 0.1)
S Na (mmol/L)	133 (± 1.3)	130 (± 1.8)	132 (± 3.7)	136 (± 5.0)*
S K (mmol/L)	4 (± 0.4)	4 (± 0.3)	4 (± 0.3)	4 (± 0.3)
U Osm (mosmol/kg)	587 (± 153.3)	623 (± 136.5)	637 (± 112.2)	690 (± 122.0)
U Urea (mg/dL)	938 (± 511.4)	1031 (± 476.8)	1806 (± 461.6)*	2187 (± 534.1)*
U Creat (mg/dL)	44 (± 25.5)	45 (± 23.0)	30 (± 15.0)	29 (± 16.3)
U Na (mmol/L)	127 (± 32.9)	139 (± 43.2)	112 (± 44.0)	93 (± 39.0)
FE.Na (%) N < 1.5%	1.2 (± 0.6)	1.2 (± 0.5)	1.51 (± 0.9)	1.31 (± 0.6)
FE.Osm (%) N < 3%	2.38 (± 0.6)	2.45 (± 0.5)	3.99 (± 1.8)*	4.62 (± 1.6)*

*P < 0.01 compared to day 0.

S, serum; U, urine; FE.Na, fractional sodium excretion; FE.Osm, fractional osmolytes excretion.

To convert S urea (mg/dl) in BUN (mg/dl) multiplied by 0.467.

Pharmaceutical grade urea (medicinal urea, Certa^®^, Braine l'Alleud, Belgium) is usually prepared by the pharmacy in bags of 15 or 30 g [22], which are dissolved in 100 ml water and given by gastric tube over 5 to 10 minutes (except in case of brain haemorrhage in which urea is given continuously). In some patients urea is directly dissolved in the liquid nutritional support. Patients taking it orally dissolved urea in orange juice (or syrup) and take it after the meal.

### Study II - Severe hyponatremia (≤ 115 mmol/L)

**Figure 2:**
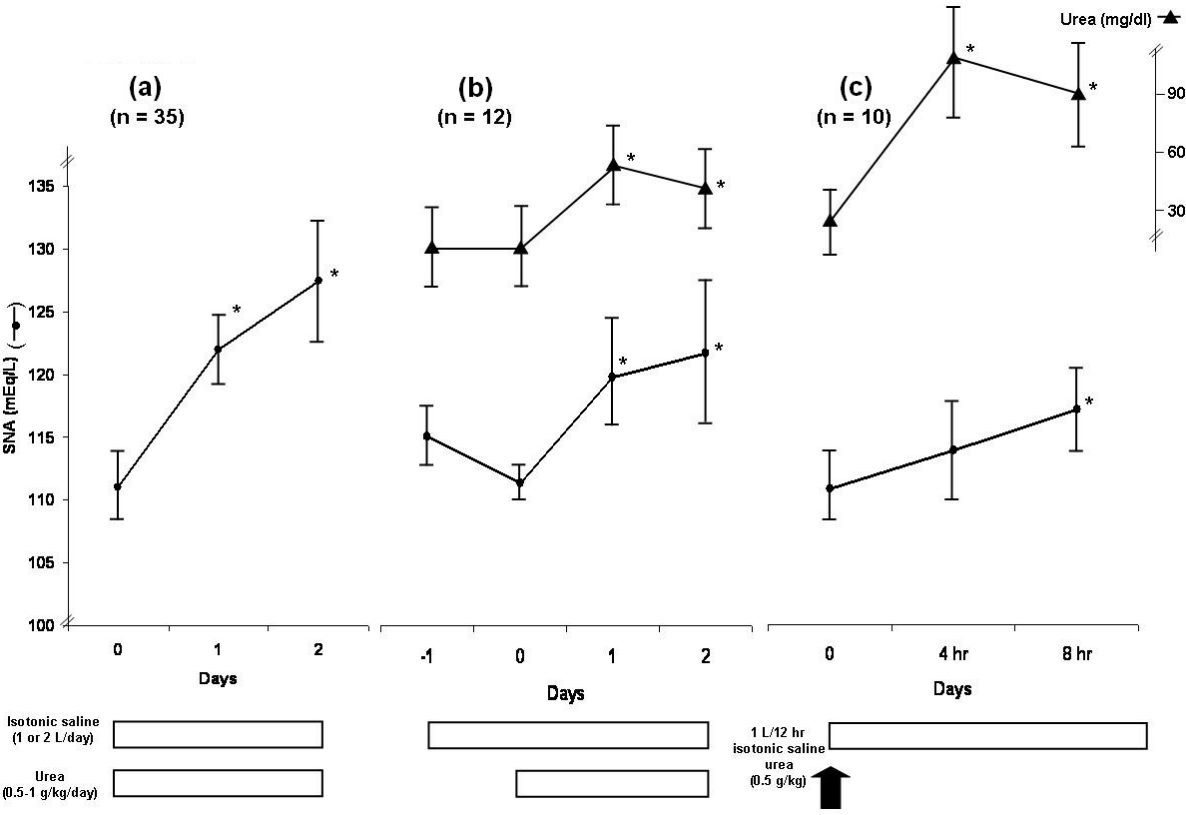
**Evolution of SNa in 35 patients with severe hyponatremia treated by urea and isotonic saline**. **(a) **Daily evolution of SNa (mean ± SD) in 35 patients with severe hyponatremia (≤ 115 mEq/L) treated with isotonic saline and urea (*P < 0.001 compared to Day 0). **(b) **Daily evolution of SNa (mean ± SD) in 12 patients with severe hyponatremia (≤ 115 mEq/L) who received the first day only isotonic saline (1 or 2 L). The addition of urea increased significantly SNa (*P < 0.01 compared to Day 0). **(c) **Evolution of SNa (mean ± SD) each four hours in 10 patients with severe hyponatremia treated with 1 L isotonic saline over 12 hr and urea (0.5 g/kg) (*P *< 0.01 compared to time 0).

We analyzed the records of 35 consecutive patients with severe hyponatremia treated with urea (Figure 2a).

In 12 of these patients urea was added 24 hr after 1 or 2 L isotonic saline (Figure 2b).

In 10 patients, SNa and S urea were measured before and every 4 hr after urea (0.5 g/kg/12h) administration combined with perfusion of 1 L isotonic saline each 12 hr (Figure 2c).

In all the patients SNa was measured at least two times (a few hours to 24 hrs before urea therapy).

For Studies I and II, an ethical approval was obtained. Data are provided as mean ± SD, we use the one-way analysis of variance and the Tukey-Kramer multiple comparisons test.

## Results

### Moderate hyponatremia

Figure 1 presents the evolution of SNa and S.urea in 50 patients treated by urea for mild hyponatremia developed it in the intensive care unit (mean age: 71 ± 20 years). Two-thirds of the patients were receiving isotonic saline (1 or 2 L/day) and one-third received half isotonic saline (1 or 2 L) during the two days before and after urea administration. It is usual in our intensive care unit that when infusion induced or aggravated hyponatremia to add urea, while maintaining the same volume of liquid administration (liquid nutritional support, medication, and so on).

SNa increased significantly in all the patients (ΔSNa in two days: 7 ± 4 mEq/L; *P *< 0.001)). The dose of urea varied between 15 and 120 g/day given usually by gastric tube in one to four doses. The mean dose was 46 ± 25 g/day. Ten patients received 15 g/day, 10 received 30 g/day, 11 received 45 g (usually 3 × 15 g/day), 14 received 60 g/day, 3 received 90 g/day and 2 received 120 g/day. Urea was given by gastric tube in 80% of the patients and by mouth in 20%.

Table 1 presents the evolution of some blood and urine parameters in 10 patients where these data were available; all these patients received at least 45 g/day urea during three days. Table 1 shows that in these patients the intake of urea was associated, as expected, with an increase in urine urea concentration (while urine osmolality remained high). In those patients, total liquid input the first day of urea therapy was estimated at 3.031 ± 1.244 mL and output was 3,905 ± 1,016 mL (mean difference 874 mL) (*P *< 0.01) which contribute to the increase in SNa despite the high fluid intake.

The origin of SIADH was due to different brain diseases (tumour, subdural hematoma, subarachnoid haemorrhage, brain hematoma, posttraumatic brain injury, meningitis, and so on) in 80% of the patients and in 20% vs non brain diseases (neoplasms, pneumonia, COPD, and so on). Seven patients developed hypernatremia during urea therapy (maximum value of SNa 155 mEq/L).

The dose of urea was adjusted by the intensivists depending of the increase in SNa.

Mean duration of treatment was six days (from 2 to 42 days in the ICU). Hyponatremia recurred in six patients when urea was stopped, which necessitated its reintroduction.

### Severe hyponatremia

Figure 2a presents the evolution of SNa in 35 patients with severe hyponatremia which was acquired outside the hospitals (in 10 patients it was due to thiazides, in 8 to neoplasic diseases, and so on). Most patients presented neurological symptoms (four were asymptomatic, four were comatose, three presented seizures, all the other were described as confused or somnolent). SNa increased from 111 ± 3 mEq/L to 122 ± 4 mEq/L in one day (*P *< 0.001) and all the patients with neurological symptoms made a rapid recovery.

SNa increased more than 12 mmol/L the first day in 12 patients and in 13 patients the increase in SNa was higher than 18 mmol/L/48 hr. In two of these patients the intensivist lowered the SNa again by giving desmopressin (DDAVP) and water. No cases of clinical osmotic demyelination syndrome (ODS) developed. When high doses of urea are used (≥ 60 g/day) it is usual to avoid the next dose of urea if blood urea level is higher than 150 mg/dL (SNa and urea are measured in most patients every 8 to 12 hr when large doses are used). No cases of hypernatremia were observed. The 10 patients with thiazides induced hyponatremia presented biological data similar to patients with SIADH (low urea and uric acid levels) [23,24]; it is likely that isotonic saline alone was sufficient in most of these patients.

Seven patients presented hypokaliemia (range 2.3 to 3.4 mmol, four were taking diuretics).

All the patients presented with a systolic blood pressure over 100 mmHg and showed no signs of overt hypovolemia (clinically or biologically).

In 12 patients (Figure 2b) urea therapy was initiated after 1 or 2 L isotonic saline (given over one day) and which did not improved natremia (two were on thiazide).

In 10 patients (Figure 2c), 1 L isotonic saline was administered each 12 hr with 0.5 g/kg of urea (given by mouth or gastric tube). In these patients, mean SNa increased by 7 ± 4 mmol/L in eight hours (range 1 to 11 mmol/L; *P *< 0.01). Urea increased from 27 ± 14 mg/dL to 96 ± 30 mg/dL four hours after urea administration (*P *< 0.001).

## Discussion

Our data show that urea is an efficient and safe method to manage hyponatremia in the intensive care unit. Urea has been used orally or intravenously over time as an osmotic diuretic drug and as an agent to reduce intracranial and intraocular pressure [25]. As opposed to mannitol, urea enters intracellular spaces rapidly (in less than one hour) throughout the body, decreasing the immediate risk of sudden cardiac decompensation due to rapid intravascular volume expansion and does not induce a transient decrease in SNa as observed with mannitol (translocation hyponatremia) [26]. However, because urea penetrates into the CNS only about one-tenth as quickly as into muscle, a significant intravascular to CNS urea gradient occurs (during 4 to 10 hrs) [27]. Decreases in brain water content and intracranial pressure during urea administration have been measured experimentally and/or clinically [25].

In this study, urea was not used to treat eventual brain oedema due to hyponatremia [28,29].

High doses of urea can be given on a long-term basis without renal toxicity which is not the case for mannitol. In individuals with previously normal baseline renal function, the mean total dose of mannitol that precipitated acute renal failure was 626 ± 270 g over two to five days [30] (which represents about 209 g ± 90 g urea on an equimolar basis).

Many patients presented hyponatremia associated with various brain diseases, it is likely that most presented SIADH as isotonic saline infusion (2/3 of the patients) or half-isotonic saline (1/3 of the patients) was not able to correct SNa [31], while the introduction of urea corrected SNa (Figures 1 and 2b).

The mean increase in SNa was around 4 mmol/L the first day of urea administration and 7 mmol/L in 48 hrs in the patients with mild hyponatremia, these results are similar to those reported with conivaptan whether used orally or intravenously [10-16]. In our study there was no fluid restriction, while in the conivaptan studies fluid intake was less than 2 L/day. Conivaptan is a substrate and potent inhibitor of the microsomal enzyme cytochrome P450 (CYP) 3A4, the concomitant use of several agents are prohibited, including chemotherapeutic agents, calcium channel blockers, 3-hydroxy-3 methyl-glutaryl-coenzyme A reductase inhibitors, benzodiazepines, and immunosuppressants. Use of conivaptan has been allowed for four-days treatment and by the intravenously route. Another anti V2 medication need to be used for patients with persistent SIADH (which is frequent) while urea has no long-term toxicity [25,32,33] and can also be used intravenously, although in the present study the 85 patients with hyponatremia were all treated orally (less expensive than the intravenous route; for example Ureaphil where each bottle of 135 mL contains 40 g urea which need to be dissolved in 5% dextrose) [19,22]. In the large SALT trials with Tolvaptan, patients had free access to water but first day of treatment SNa increases of about 2 to 3 mmol/L and about 7 mmol/L at the end of the study on Day 30 [17]. We did not include in this study patients with symptomatic acute hyponatremia (mainly postoperative). All our patients with severe hyponatremia developed it outside the hospital and are considered as at least partially, chronically hyponatremia (> 48 hr) and no patients presented with primary polydipsia.

In this series of severe hyponatremic patients, only four were frankly comatose but the majority were symptomatic and were treated by isotonic saline combined with urea given orally (by gastric tube if needed). In our hospital severe euvolemic hyponatremia is usually treated with a combination of urea and isotonic saline which is an alternative to hypertonic saline [21]. At the present time, severe symptomatic hyponatremia particularly if epileptic seizures are present should be treated with hypertonic saline (consensus conference) [34]. Hypertonic saline will increase SNa theoretically more rapidly than urea.

Establishment of a depletional origin of hyponatremia is not always easy particularly in the medical ward [35,36]. The combined treatment of isotonic saline and urea has some advantages. If there is some salt depletion, isotonic saline will be useful while if excess water is the main factor of hyponatremia, urea will be useful (by subtracting water).

Although we discourage an increase of SNa of more than 10 mmol/L/day, many patients presented a too large increase [21,22,34]. We know that urea has a protective effect against osmotic demyelination syndrome (ODS) in animals [37-40]. SNa was decreased again only in two patients by giving DDAVP and water [41-44]. No clinically cases of ODS were observed in our patients, this could reflect the protective effect of urea.

In all the studies published with the vaptans, no patients with SNa less than 115 mEq/L where included. Despite the attractiveness of using a pure aquaretic agent to correct life-threatening hyponatremia, insufficient data are available from clinical trials to know if sufficiently rapid correction can be achieved in patients with acute, severe hyponatremia without the use of hypertonic saline. Indeed, present studies show that V2RA diuresis does not begin to increase before one to two hours.

Urea in large doses (1 g/kg), when administered rapidly by gastric tube in a matter of minutes, has a purgative effect which creates troublesome nursing problems in the comatose patients. This effect could be avoided by administering the urea over a long period of time, or by fractioning the dose. In our study, this was never a problem; no tracheal aspiration was reported, but we avoided giving urea rapidly in large amounts. We also administered urea continuously (for example in the liquid nutritional support) in patients with brain haemorrhage, to avoid any brain shrinkage. As previously mentioned, urea was not used to treat brain oedema in this study (although this could represent a good indication, particularly if the intravenous route is used) [21]. We can expect that acute administration of urea at 0.5 gr/kgBW intravenously over one hour or orally will rapidly (one hour) increase serum osmolality by 15 mOsm/kg/H_2_O during a few hours (renal elimination).

These data report the use of urea in an intensive care unit, but urea can also be used to treat many patients over the long-term (years) without problems and likely with similar efficacy than the V2 antagonists but at a much lower price [45]. In many patients, taste is not a complaint, particularly if low doses are sufficient to control hyponatremia (15 to 30 g/day).

## Conclusions

These data emphasise that urea combined with isotonic saline is an easy way to treat euvolemic hyponatremia in the ICU.

A prospective treatment comparing this old treatment with the V2RA needs to be done.

## Key messages

• In the intensive care unit, urea combined with isotonic saline is an easy and inexpensive way to treat euvolemic hyponatremia.

## Abbreviations

DDAVP: desmopressin; FE.Na: fractional excretion of sodium (in %); FE.osm: fractional excretion of osmoles (in %); ODS: osmotic demyelination syndrome; SIADH: syndrome of inappropriate secretion of ADH; SNa: serum sodium; V2RA: V2 receptor antagonist.

## Competing interests

The authors declare that they have no competing interests.

## Authors' contributions

All the authors contributed to the collection and analyse of the data in a similar way. All authors read and approved the final manuscript.
